# The Emerging Role of Exosomes in Epithelial–Mesenchymal-Transition in Cancer

**DOI:** 10.3389/fonc.2014.00361

**Published:** 2014-12-19

**Authors:** Laura Jayne Vella

**Affiliations:** ^1^Ludwig Institute for Cancer Research, Melbourne-Austin Branch, Cancer Immunobiology Laboratory, Olivia Newton-John Cancer and Wellness Centre, Heidelberg, VIC, Australia; ^2^The Florey Institute for Neuroscience and Mental Health, Parkville, VIC, Australia

**Keywords:** exosomes, cancer, extracellular vesicles, intercellular signaling

## Abstract

Metastasis in cancer consists of multiple steps, including epithelial–mesenchymal-transition (EMT), which is characterized by the loss of epithelial-like characteristics and the gain of mesenchymal-like attributes including cell migration and invasion. It is clear that the tumor microenvironment can promote the metastatic cascade and that intercellular communication is necessary for this to occur. Exosomes are small membranous vesicles secreted by most cell types into the extracellular environment and they are important communicators in the tumor microenvironment. They promote angiogenesis, invasion, and proliferation in recipient cells to support tumor growth and a prometastatic phenotype. Although it is clear that exosomes contribute to cancer cell plasticity, experimental evidence to define exosome induced plasticity as EMT is only just coming to light. This review will discuss recent research on exosomal regulation of the EMT process in the tumor microenvironment.

## Introduction

Epithelial–mesenchymal-transition (EMT) is a process whereby epithelial cells undergo a shift in plasticity and acquire the ability to disseminate, invade, and cause metastasis. Established as a central process during the early stages of development, it is now clear that EMT has implications on cancer progression by triggering the loss of cell–cell adhesion to facilitate tumor cell invasion and remodeling of the extracellular matrix. While epithelial cells express high levels of E-cadherin and are closely connected to each other by tight junctions, mesenchymal cells express N-cadherin, fibronectin, and vimentin, have a spindle-shaped morphology and less tight junctions.

Intercellular crosstalk between neighboring and distant tumor cells and immune and stromal cells in the tumor microenvironment plays a large role in cancer development, the establishment of the mesenchymal state, and metastasis. Intercellular crosstalk can occur by direct cell to cell contact or via factors secreted into the extracellular environment. Extracellular vesicles, called exosomes, have become recognized as important in cellular communication (1). Unlike soluble factors secreted by cells, exosomes carry a concentrated group of functional molecules, provide protection to the transported molecules and serve as intercellular communicators not only locally but also systemically.

Exosomes are formed from inward budding of the limiting membrane of multi-vesicular bodies (MVB) and are released from the cell into the extracellular environment upon fusion of the MVB with the plasma membrane. Most prokaryotic and eukaryotic cells release exosomes, including cancer cells such as colorectal (2), lung, breast, glioblastoma (GBM), ovarian, and melanoma (3). Exosomes from different cellular types contain a common set of molecules, as well as cell type-specific components. For example, exosomes derived from cancer cells contain proteins that reflect the endosomal origin of exosomes as well as cellular oncogenic drivers including receptor tyrosine kinases (RTKs), oncoproteins, phosphorylated proteins, and miRNA (2, 4–6). After release into the extracellular environment, exosomes act as discrete vesicles trafficking to distant and proximal recipient cells where they alter cell signaling and phenotype by transfer of bioactive molecules. Exosomes transfer their messages in different ways. Firstly, they can activate target cells through the transfer of ligands such as fibroblast growth factor (FGF), hepatocyte growth factor (HGF), vascular endothelial growth factor (VEGF) (7, 8), and epidermal growth factor (EGF) (9). Secondly, they can transfer receptors such as mutant EGFR (10) and HGFR (11) from one cell to another by fusion with the plasma membrane of recipient cells (10). This results in transfer of oncogenic activity via activation of growth factor signaling pathways in recipient cells (11, 12). The third mechanism of action involves endocytosis of the exosome and subsequent transfer of molecules directly into the cytosol of the recipient cell. These can include phosphorylated P13K, AKT, mTOR, cyclins, and cyclin-dependent kinases (13, 14) and miRNA, which can functionally repress target genes in the recipient cell (15).

Over the last decade, a number of studies have demonstrated that exosomes are mediators of the metastatic process. Exosomes derived from both normal and cancer cells can promote angiogenesis (16–19), invasion (20–23), and proliferation (24–26) in recipient cells to support tumor growth.

## Changes in Exosome Composition Accompany the Transition to a Mesenchymal State

Epithelial–mesenchymal-transition entails morphological and phenotypic changes to a cell. To assess the composition of exosomes released from cells following these changes, several groups have induced EMT via transformation with oncogenic proteins such as Ras or EGFR (27–29). Exosomes released from Madin–Darby canine kidney (MDCK) cells transformed with oncogenic H-Ras contained the EMT marker vimentin, in addition to matrix metalloproteases (MMPs), integrins, and key and core splicing complex components (29). Epithelial markers including E-cadherin and EpCAM were downregulated relative to exosomes from untransformed cells. It was postulated that exosomes from the transformed cells were capable of inducing EMT in recipient cells although no functional experiments were performed to validate this. Proteomic studies on EGFR (coupled with blockade of E-cadherin) induced EMT in A431 and DLD-1 epithelial cancer cells, revealed coordinated loss of EGFR and tissue factor (TF) from the cells (27). This coincided with an increase in exosome release, selective upregulation of TF in exosomes, and expression of 30 additional proteins unique to the mesenchymal cell-derived exosomes (28). The mesenchymal-like cells transferred TF to recipient endothelial cells via exosomes rendering the recipient cells procoagulant, suggesting EMT promotes exosome release and shedding of TF from cells via exosomes (27).

Jeppesen et al. studied the protein content of exosomes derived from a human bladder carcinoma cell line without metastatic capacity relatively to two isogenic derivate metastatic cell lines formed in the lung and liver of mice. Although proteins associated with EMT were found in exosomes derived from the metastatic cells (30), no functional studies correlating changes in protein content with alterations in exosome function were carried out, so it is unclear in this case if exosomes from the metastatic cell line had an increased metastatic potential. With that said, exosomes from a range of mesenchymal-like breast and ovarian cancer cell lines differentially impacted on recipient cells compared to epithelial-like cell lines (31). Exosomes from the mesenchymal-like cell lines contained increased angiogenic molecules including PDGF, IL-8, and angiogenin suggested to promote AKT phosphorylation and subsequent activation of recipient endothelial cells (31).

## EMT Inducers are Associated with Exosomes

The protein composition of exosomes has been analyzed extensively, predominantly by mass spectrometry to reveal a defined subset of cellular proteins common to exosomes originating from a variety sources and species (32–35). Inducers of EMT have been found in association with exosomes including TGFβ (36), TNFα, IL-6, TSG101, AKT, ILK1, β-catenin (37, 38), hepatoma-derived growth factor, casein kinase II (CK2), annexin A2 (30), integrin 3 (39), caveolin-1 (40), and matrix metalloproteinases (41–44). Functional studies to demonstrate that exosome associated EMT inducers promote a prometastatic phenotype are outlined below.

The WNT signaling pathway participates in EMT by inhibiting glycogen synthase kinase-3β (GSK3β) to stabilize β-catenin, promoting a gene expression program that favors EMT (45). Exosomes released from human and *Drosophila* cells contain WNT (46, 47), which can be transferred and activate WNT signaling in recipient cells (48–50). Luga et al. observed that WNT containing exosomes derived from cancer associated fibroblasts (CAFS) promoted motility and metastasis by activating autocrine WNT-planar cell polarity signaling in recipient breast cancer cells (48). Similarly, mesenchymal stem cell (MSC) and macrophage-derived exosomes (51) promoted migration and/or invasion of breast cancer via activation of WNT signaling (49). In melanoma, recombinant WNT5A induces the release of soluble mediators including IL-6, IL-8, VEGF, and MMP2 in association with exosomes (52) suggesting that not only does exosomal WNT promote EMT in recipient cells but it changes the composition of the released exosome to promote further EMT. Kock et al. examined the contribution of exosomes to cancer population equilibrium and tumor heterogeneity (53). They showed that diffuse large B-cell lymphomas possess a self-organized infrastructure comprising two populations of cells, where transitions between clonogenic states could be modulated by exosome-mediated WNT signaling (53). This study goes some way in broadening our understanding of the complex processes that maintain tumor cell heterogeneity and highlights exosomes as key players in this process.

Hypoxia in the tumor environment can promote EMT and several studies have provided evidence that hypoxia promotes the release of exosomes from different tumor cell types including breast, glioma, leukemia, and prostate (38, 54–57). Exosomes released by prostate cancer cells under hypoxic conditions contain more TGFβ IL-6, TNFα, and MMP, TSG101, AKT, ILK1, and β-catenin (38), suggesting that they could differentially modulate recipient cells compared to exosomes from normal cells. Indeed, exosomes released from A431 carcinoma (58), glioma cells (55), and leukemia cells (54) promoted angiogenesis in recipient cells (16, 55). Similarly, exosomes derived from hypoxic GBM cells promoted tumor cell survival by inducing angiogenesis both *in vitro* and *ex vivo* through phenotypic modulation of endothelial cells and increased autocrine, promigratory activation of GBM cells (57).

Latent membrane protein 1 (LMP) of Epstein–Barr virus (EBV) contributes to the metastatic phenotype of nasopharyngeal carcinoma (NPC) by inducing EMT. Aga et al. (22) investigated if LMP1-positive exosomes could mediate EMT. They demonstrated that LMP1 positive exosomes and exosomal HIF1α modulate expression of EMT markers in recipient cells (22). Following treatment with LMP1-positive exosomes, recipient cells expressed less E-cadherin and more N-cadherin along with morphological spindle-like changes in cell shape indicative of EMT (22). Although exosome concentration was not reported and downstream signaling pathways associated with EMT were not examined, it is clear that LMP1-positive exosomal transmission of HIF1α correlates with EMT-associated changes in the cadherin expression profile in recipient cells.

A growing number of miRNAs have been implicated in the regulation of EMT-related pathways in cancer (59) and in recent years exosomes have been reported to contain nucleic acid such as DNA, RNA, non-coding RNA, and miRNA (60–62). MiR-223, a miRNA specific for IL-4-activated macrophages, could be transported from macrophages to breast cancer cells via exosomes (63) to promote breast cancer cell invasion via modulation of the β-catenin pathway. Similarly, exosomes released from bone marrow-derived mesenchymal cells promoted multiple myeloma (MM) formation in an animal model by transfer of exosomal miR-15a (64). Josson et al. recently performed one of the first studies to show that transfer of stromal-derived exosomal miRNA results in morphologically and biochemically defined EMT in cancer cells (65). Exosomes were isolated from normal prostate stromal cells overexpressing miR-409. Exosome associated miR-409-3p and -5p decreased the expression of target genes in prostate cancer cells and increased proliferation. Interestingly, 6 weeks after maintaining the prostate cancer cells in stromal cell media, the prostate cancer cells underwent EMT, which was biochemically defined by decreased E-cadherin and increased vimentin mRNA expression. *In vivo*, co-injection of prostate cancer cells and miR-409-overexpressing stromal fibroblasts resulted in tumor cells expressing miR-409 and enhance tumor growth suggesting that miR-409 was secreted out of stromal fibroblasts and taken up by the adjacent tumor. Further *in vivo* modeling however is required to conclude that stromal-derived exosomes were responsible for transfer of miR-409 to surrounding cancer epithelial cells and subsequent tumor growth.

## Exosomes Released from Tumor Cells Promote Phenotype Change in Stromal Cells

The tumor microenvironment consists of a complex network consisting of an extracellular matrix populated by CAFs, endothelial cells, and immune cells. Exosomes derived from tumor cells communicate with stromal cells and vice-versa to promote tumor growth. MSCs have multi-lineage potential and can differentiate into a variety of cell types including tumor stromal cells, which are pro-tumorigenic. One way they do this is by promoting differentiation of MSCs, in some cases via mesenchymal-to-epithelial transition (MET).

Ovarian cancer cell-derived exosomes can induce adipose tissue-derived MSCs (ADSC) to exhibit the characteristics of CAFs, by increasing expression of TGFβ and activation of Smad-dependent and -independent pathways (9). Similarly, gastric cancer exosomes trigger differentiation of umbilical cord-derived MSCs to CAFs through the TGFβ/Smad pathway (66) and breast and prostate cancer-derived exosomes can induce a myofibroblastic phenotype (67, 68). Together, these studies show that via activation of both Smad-dependent and -independent pathways, tumor-derived exosomes can hijack MSCs to promote a prometastatic environment. In some cases, this process appears dependent on TGFβ1 expressed at the exosome surface in association with the transmembrane proteoglycan betaglycan (67). Although existing in a latent state, this complex was fully functional in eliciting Smad-dependent signaling in recipient cells. Interestingly, myofibroblasts generated using soluble TGFβ1 were not pro-angiogenic or tumor-promoting, suggesting that exosomal TGFβ1 is required for the formation of tumor-promoting stroma (36).

In an elegant series of experiments, Abd Elmageed et al. demonstrated that tumor-tropic patient-derived ADSCs primed with prostate cancer cell-derived exosomes undergo genetic instability, MET, oncogenic transformation, and develop prostate tumors *in vivo* (69). Oncogenic transformation was associated with down-regulation of tumor suppressors upon delivery of prostate cancer-derived exosomal oncogenic H-ras and N-ras transcripts, Rab proteins, and oncogenic miRNA.

## Conclusion

Exosomes play an important role in the development and progression of cancer. The studies outlined above highlight their role in the regulation of EMT-related pathways and suggest that tumor and stromal cells can regulate the invasiveness of cancer cells through exosome-mediated delivery of protein and miRNA. In the last decade, there has been an exponential increase in the number of studies aiming to understand the biology and composition of exosomes. These studies established that exosome composition changes upon transition to a mesenchymal state and that EMT inducers are associated with exosomes. In the last 2 years, experimental evidence has come to light defining exosome induced plasticity in recipient cells as EMT. Future investigations should further reveal how multiple cellular populations communicate via exosomes to promote a premetastatic phenotype and how exosomes can be employed for diagnostic and prognostic purposes to improve patient outcome.

## Conflict of Interest Statement

The author declares that the research was conducted in the absence of any commercial or financial relationships that could be construed as a potential conflict of interest.
